# Androgen receptor/let-7a signaling regulates breast tumor-initiating cells

**DOI:** 10.18632/oncotarget.23196

**Published:** 2017-12-08

**Authors:** Wei Zhang, Xiaozhen Liu, Shan Liu, Ying Qin, Xiaoqi Tian, Fengting Niu, Han Liu, Ning Liu, Yun Niu

**Affiliations:** ^1^ Department of Breast Cancer Pathology and Research, National Clinical Cancer Research Center, Key Laboratory of Cancer Prevention and Therapy, Tianjin Medical University, Tianjin, China; ^2^ Department of Anatomy and Histology, School of Basic Medical Sciences, Tianjin Medical University, Tianjin, China; ^3^ Laboratory of Epigenetics and Tumorigenesis, Tianjin Research Center of Basic Medical Sciences, Tianjin Medical University, Tianjin, China; ^4^ Department of Pathology, Baodi Clinical Institute of Tianjin Medical University, Tianjin, China

**Keywords:** breast cancer, androgen receptor, let-7a, tumor-initiating cells, CD44^+^/24^-/low^

## Abstract

Androgen receptor (AR) is an important transcriptional factor, which is frequently expressed in invasive breast cancer and correlates patients’ prognosis. Our previous results indicate AR activation may increase let-7a expression in breast cancer cells, while let-7, a tumor suppressor, is reported to inhibit breast tumor-initiating cells (T-IC). The study aims to explore the effects of AR/let-7a signaling on breast T-IC and its regulatory mechanism. The results revealed that the expression of AR was significantly associated with let-7a and CD44^+^/24^-/low^ especially in estrogen receptor positive (ER+) breast cancer tissues. The expression of AR and let-7a indicated better outcome, while patients with CD44^+^/24^-/low^ phenotype had worse prognosis. AR activation induced by dihydrotestosterone (DHT) prevented cells proliferation, migration, invasion and self-renewal capacities in ER+ breast cancer cells, via transcriptional up-regulation of let-7a. In addition, AR could inhibit tumorigenesis and metastasis of ER+ breast cancer cells in the serial xenotranplanted animal models. Our data suggested that AR/let-7a signaling could inhibit the biological behavior of tumor-initiating cells (T-IC) in ER+AR+ breast cancers, which might become a new therapeutic target.

## INTRODUCTION

Androgen receptor (AR) is frequently expressed in invasive breast cancers and may be a diagnostic and therapeutic target for breast cancer [1–3]. It is recently indicated tumor-initiating cells (T-IC), a small subset in cancer that has the capacities of self-renewal, mutipotent differentiation and tumorigenicity, may be affected by AR activation in prostate cancer, contributing to the progression of the malignant tumors [4–7]. AR is considered as a significantly prognostic factor in triple-negative breast cancer, which is enriched in T-IC [8]. Breast T-IC can be enriched by sorting for CD44^+^/CD24^-/low^ cells [7], by selecting for side-population cells that efflux Hoechst dyes [9], or by isolating spherical clusters of self-renewing cells (“mammospheres”) from suspension cultures [10]. However, little information is known about the correlations between AR and breast T-IC capabilities in breast cancers.

As a critical transcriptional factor, AR regulates the transcription of various target genes, including MicroRNAs (miRNAs) [11]. MicroRNAs are endogenous, small, non-coding RNAs that interfere with protein expression by inducing cleavage of their specific target transcripts or by inhibiting their translation, through complementing the 3’ untranslated regions in the target transcripts [12]. Let-7 is the earliest discovered miRNAs and encodes an evolutionarily conserved family of 13 homologous miRNAs frequently deleted in variety of human cancers [13]. Let-7a, one of the members of let-7 family, is usually selected as the representative [14, 15]. In addition, lack of let-7a is necessary for tumorigenicity and self-renewal of breast T-IC, while let-7a overexpression not only prevents cell proliferative and self-renewal capacities, but also converts higher metastatic T-IC into lower malignant cells [16].

We previously found AR activation induced a robust increase of let-7 family members in breast cancer cell lines, including let-7a [11]. We hypothesize that there are some correlations among AR, let-7a and BT-IC, and they maybe co-regulate the progression of breast cancer. The present study aims to examine the expression of AR, let-7a and CD44^+^/CD24^-/low^ in invasive breast cancer tissue specimens. And the effects of AR/let-7a signalling on the stem-like behavior of breast cancer cells are also evaluated *in vitro* and *in vivo*. We may try to elucidate the mechanism of AR regulating let-7a.

## RESULTS

### AR was correlated with BT-IC and let-7a in breast cancer tissue specimens

**Figure 1:**
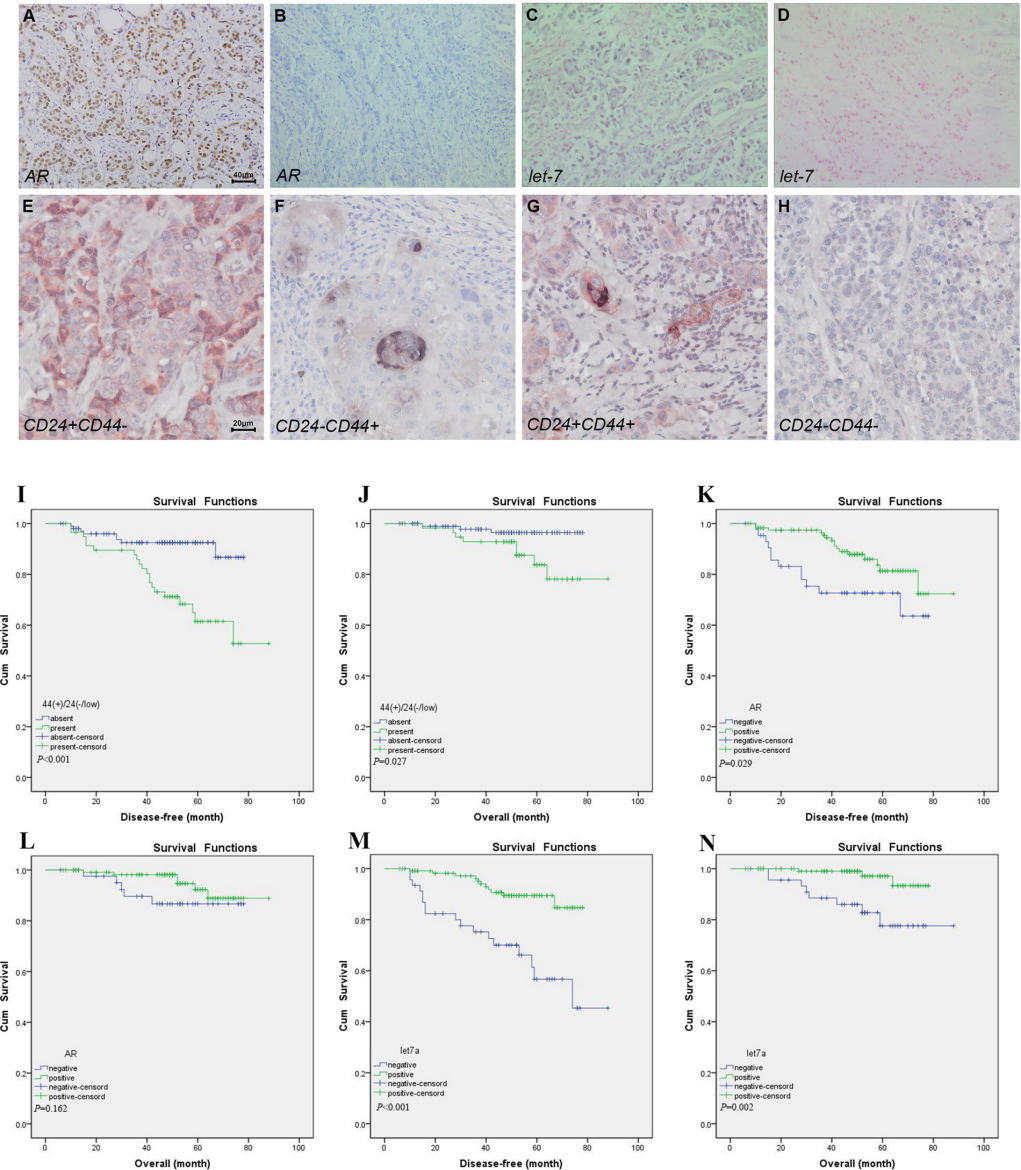
Expression of AR, CD44/CD24 and let-7a and prognosis Immunohistochemical staining of AR positive. (**A**) and the negative control (**B**), original magnification ×200. ISH of let-7a positive (**C**) and the negative control (**D**), original magnification ×200. Double immunohistochemical staining of CD44^-^/CD24^+^ (**E**), CD44^+^/24^-/low^ (**F**), CD44^+^/CD24^+^ (**G**) and CD44^-^/CD24^-^ (**H**), original magnification ×400. DFS and OS curves about CD44^+^/24^-/low^ (**I** and **J**), AR (**K** and **L**) and let-7a expression (**M** and **N**) status in 165 patients with invasive breast carcinoma.

CD44 and CD24 expression was evaluated by double-staining immunohistochemistry technique in 165 cases of breast cancer tissues, and the cancer cell with CD44^+^/CD24^-/low^ phenotype might be considered as BT-IC. CD44 showed membranous and/or cytoplasmic staining in black color with 5-bromo-4-chloroindo-3-yl-phosphate/nitro blue tetrazolium (BCIP/NBT) substrate, and CD24 showed membranous and/or cytoplasmic staining identified by red deposits with Peroxidase-3-amino-9-ethylcarbazole (AEC) response. CD44^+^/24^-/low^ tumor cells was determined as the cells positive for black color but negative for red staining. CD44^+^/24^-/low^ tumor cells ≥1% was considered positive as previously reported [17]. AR positivity was defined as ≥ 10% of tumor cells presented nuclear staining with different degrees. Let-7a was considered positive if 10% cytoplasmic and/or membranous staining was observed as indigo blue color. As indicated by Figure 1, there were 59 (35.8%) cases with CD44^+^/CD24^-/low^ phenotype, 121 (73.3%) with AR positive and 118 (71.5%) with let-7a positive. The expression of AR was positively correlated with CD44^+^/CD24^-/low^ presence (r = 0.336, *P* < 0.001) and let-7a expression (r = 0.227, *P* = 0.003) (Table 1). Interestingly, the presence of CD44^+^/CD24^-/low^ and expression of AR were all correlated with ER expression (*P* = 0.001, *P* = 0.011, respectively, Table 2). CD44^+^/CD24^-/low^ phenotype was found in 1, 35, 3 and 20 tumors with ER+/AR-, ER+/AR+, ER-/AR- and ER-/AR+ expression, respectively. In addition, the presence of CD44^+^/CD24^-/low^ was closely associated with lymph node status (*P* < 0.001), tumor size (*P* < 0.001), progesterone receptor (PR) expression (*P* = 0.016), Ki67 (*P* < 0.001), and different molecular subtypes (*P* < 0.001); AR expression was correlated with histological grade (*P* = 0.023) and different molecular subtypes (*P* = 0.028); Let-7a was correlated with lymph node status (*P* = 0.002), pTNM stage (*P* = 0.016), Ki67 (*P* = 0.002), and different molecular subtypes (*P* = 0.021, Table 2). Survival analysis indicated that the DFS and OS differed significantly between the patients with or without CD44^+^/CD24^-/low^ phenotype (*P* < 0.001, *P* = 0.027), and the outcome of patients with CD44^+^/CD24^-/low^ phenotype was worse than that without CD44^+^/CD24^-/low^ phenotype ( Figure 1I and 1J). Patients with AR positive expression had better DFS than those with AR negative (*P* = 0.029, Figure 1K). In addition, the positive expression of AR indicated better OS than the negative ones, but there was no significant difference (*P* = 0.162, Figure 1L). Finally, patients with let-7a positive expression had better DFS and OS than those with let-7a negative (*P* < 0.001, *P* = 0.002, Figure 1M and 1N).

**Table 1:** Correlation analysis among AR, CD44^+^/CD24^-/low^ and let-7a

	CD44^+^/CD24^-/low^		r	*P*	let-7a		r	*P*
	present	absent			positive	negative		
AR			0.336	<0.001			0.227	0.003
positive	55 (33.3%)	66 (40%)			94 (60.0%)	27 (16.4%)		
negative	4 (2.4%)	40 (24.2%)			24 (14.5%)	20 (12.1%)		

**Table 2:** Correlations of AR, CD44^+^/CD24^-/low^ and let-7a with clinicopathological and biological characteristics

Clinicopathological and biological characteristics	Total cases (N = 165)	CD44^+^/CD24^-/low^ present			AR positive			let7a positive		
		% (*N* = 59)	χ2	*P* value	% (N = 121)	χ2	*P* value	% (*N* = 118)	χ2	*P* value
Age (years)										
< 35	2	100.0 (2)	3.920	0.141	100.0 (2)	1.635	0.442	50.0 (1)	0.520	0.771
35-49	79	32.9 (26)			69.6 (55)			70.9 (56)		
> 49	84	36.9 (31)			76.2 (64)			72.6 (61)		
Histological grade										
G1	20	20.0 (4)	2.465	0.292	75.0 (15)	7.557	0.023	85.0 (17)	2.050	0.359
G2	77	37.7 (29)			63.6 (49)			70.1 (54)		
G3	68	38.2 (26)			83.8 (57)			69.1 (47)		
Tumor size (cm)										
≤ 2	50	22.0 (11)	16.386	< 0.001	80.0 (40)	4.752	0.093	80.0 (40)	3.329	0.189
2 < & ≤ 5	98	35.7 (35)			73.5 (72)			69.4 (68)		
> 5	17	76.5 (13)			52.9 (9)			58.8 (10)		
Lymph node status										
negative	73	17.8 (13)	18.362	< 0.001	75.3 (55)	0.270	0.603	83.6 (61)	9.327	0.002
positive	92	50.0 (46)			71.7 (66)			62.0 (57)		
pTNM stage										
I	24	33.3 (8)	0.168	0.919	75.0 (18)	2.389	0.303	83.3 (20)	8.307	0.016
II	99	35.4 (35)			76.8 (76)			75.8 (75)		
III	42	38.1 (16)			64.3 (27)			54.8 (23)		
ER										
negative	93	24.7 (23)	11.280	0.001	65.6 (61)	6.532	0.011	71.0 (66)	0.031	0.859
positive	72	50.0 (36)			83.3 (60)			72.2 (52)		
PR										
negative	96	28.1 (27)	5.822	0.016	68.8 (66)	2.466	0.116	69.8 (67)	0.335	0.563
positive	69	46.4 (32)			79.7 (55)			73.9 (51)		
Ki67										
< 20%	55	5.4 (3)	32.979	< 0.001	74.5 (41)	0.062	0.803	87.3 (48)	10.056	0.002
≥ 20%	110	50.9 (56)			72.7 (80)			63.6 (70)		
HER2										
negative	116	32.8 (38)	1.529	0.216	69.0 (80)	3.811	0.051	74.1 (86)	1.319	0.251
positive	49	42.9 (21)			83.7 (41)			65.3 (32)		
Molecular subtypes										
Luminal A	19	0.0 (0)	43.260	< 0.001	89.5 (17)	9.116	0.028	94.7 (18)	9.766	0.021
Luminal B	59	66.1 (39)			78.0 (46)			64.4 (38)		
HER2 overexpression	36	33.3 (12)			77.8 (28)			63.9 (23)		
Triple negative	51	15.7 (8)			58.8 (30)			76.5 (39)		

### AR decreased cell proliferation and invasion by up-regulating let-7a in ER+AR+ breast cancer cells

**Figure 2:**
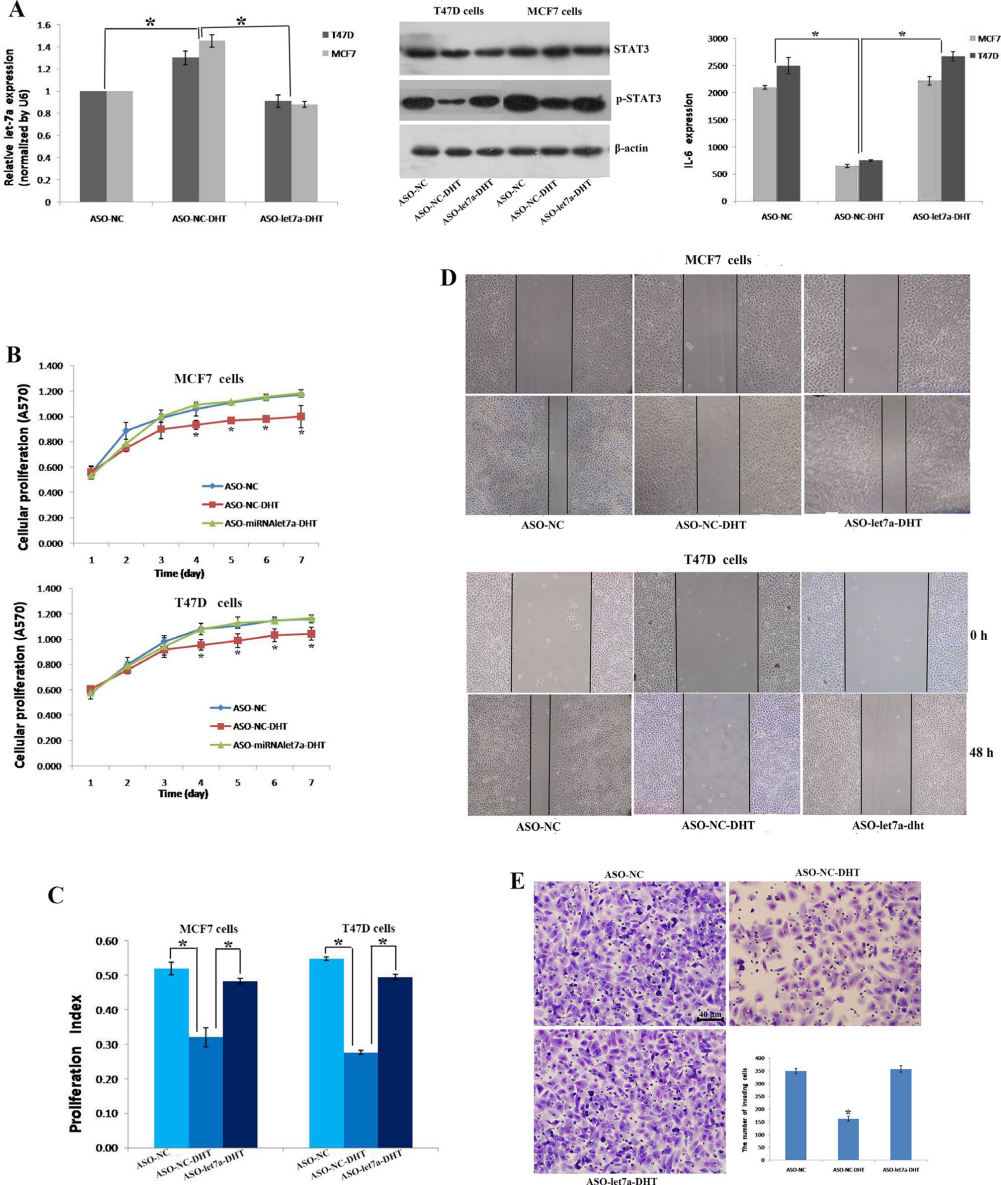
AR activation decreased cell proliferation, migration and invasion by up-regulating let-7a expression qRT-PCR showed the relative expression of let-7a in different groups of MCF7 and T47D cells (A left). Western blot showed the expression of STAT3 and p-STAT3 in T47D and MCF7 breast cancer cells (A middle). ELISA showed the secretion of IL-6 in T47D and MCF7 breast cancer cells (A right). The MTT assay was used to determine relative cellular proliferation in MCF7 and T47D cells at 1-7 days after treatment with DHT, vehicle or ASO let7a (**B**). Quantification of the cell cycle phase distribution was analyzed by flow cytometry and the proliferation index (PI) was calculated in MCF7 and T47D cells (**C**). Scratch analysis was used to detect cellular migration of MCF7 and T47D cells (**D**). The transwell assay was used to determine the invasion of T47D cells in ASO-NC, ASO-NC-DHT and ASO-let7a-DHT groups (**E**). ^*^P < 0.05.

We found more tumor cells with CD44^+^/CD24^-/low^ phenotype could be detected in ER-positive and AR-positive breast cancers (Table 2). Otherwise, few studies focused the effects of AR on let-7a expression and BT-IC properties in this subtype. Therefore, ER+AR+ breast cancer cell lines, MCF7 and T47D were selected to perform the following analysis. Results revealed that compared with control groups, let-7a expression increased had a 1.5-fold increase in MCF7 and an 1.3-fold increase in T47D cells treated with DHT, while inhibiting let-7a activity by let-7a ASO in DHT-treated cells, let-7a was significantly decreased, similar to control groups. We also analyzed the expression of STAT3 and IL-6, known target proteins of let-7a. The level of pSTAT3 and IL-6 secretion decreased when cells exposed to DHT, while no difference was found in STAT3 expression. But when let-7a expression was re-suppressed, the level of pSTAT3 and IL-6 secretion were restored at a similar level of controls (Figure 2A).

DHT treatment decreased cells proliferation, while re-lowering let-7a recovered cells growth activity, an effect similar to that illustrated without DHT treatment (Figure 2B). In addition, the cell cycle examination confirmed the results of MTT assay. When cells treated with DHT, large number of cells was prevented in the G1 phase, G1-S arrest was obvious and proliferation index was decreased. However, let-7a re-down regulation restored the cells number and cells proliferation index in the S phase, an effect similiar to that showed in the control groups (Figure 2C). DHT treatment also decreased cells healing ability, while re-knockdown of let-7a elevated cells healing ability (Figure 2D). Same results could be seen in the cells invasion. The number of T47D cells that migrated through the insert pores was 162 ± 9.54/field in DHT treated group, which was different significantly with the control group (349 ± 9.64/field) and the let-7a blocked group (357 ± 12.77/field), suggesting that DHT treatment inhibited the invasion of T47D cells, but re-knockdown of let-7a abolished the inhibited invasion (Figure 2E).

### AR decreased characteristics of self renewal of breast cancer cells

**Figure 3:**
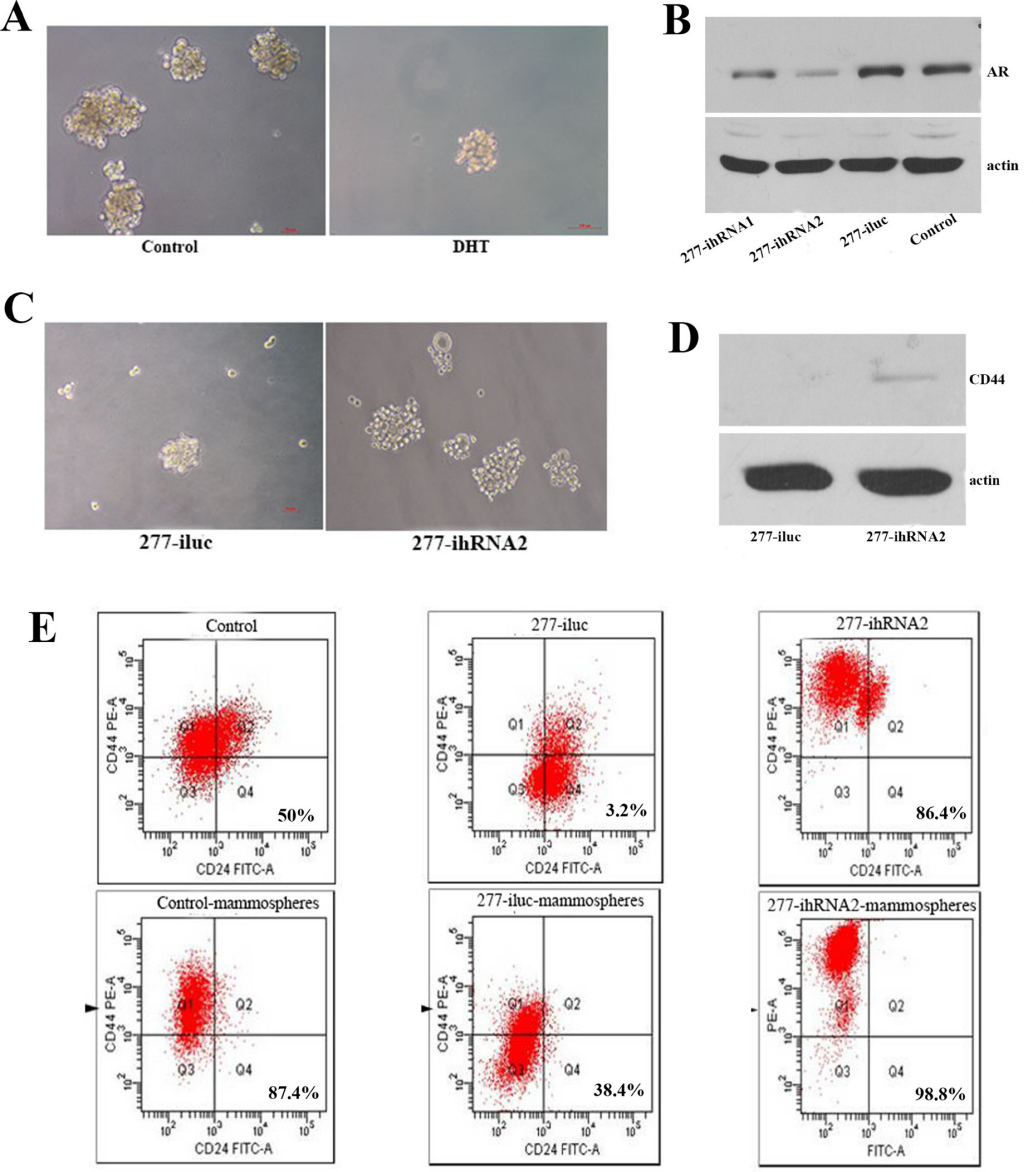
AR activation or knock-down affected characteristics of cell self-renewal of breast cancer cells Representative images showed mammospheres fromation of MCF7 with or without DHT treatment after 12 days of culture in suspension (**A**). Western blot analysis showed AR expression in MCF7 cells after being transfected with AR knockdown lentivirus or control lentivirus (**B**). After AR knock-down, MCF7 cells were cultured in suspension to generate mammospheres (**C**). Western blot analysis indicated that CD44 could be detected in AR knockdown MCF7 cells (**D**). Flow cytometry was used to detect the percentage of cells with CD44^+^/CD24^-/low^ phenotype in AR knockdown MCF7 and control cells under attachment or suspension culture (**E**).

In breast cancer tissues, AR and let-7a expression were correlated with the phenotype of CD44^+^/CD24^-/low^. Quantitative RT-PCR result showed that the up-regulation of let-7a expression induced by AR was more obvious in MCF7 than in T47D cells (Figure 2A). So MCF7 cell line was selected to analyze its capacity of self-renewing when AR was activated or AR expression was inhibited. BT-IC could be enriched by means of isolating spherical clusters of mammospheres from suspension cultures [10]. First, MCF7 cells treated with DHT were cultured in suspension to generate mammospheres. After 12 days, 1.60% ±0.36 of tumor cells formed mammospheres in DHT treated cells as compared with 3.07% ± 0.25 in the control group (*P* = 0.001, Figure 3A). AR expression was distinctly decreased in MCF7 cells expressing shRNA against AR by lentivirus, especially in 277-ihRNA2 transfected group (Figure 3B). Therefore, we selected MCF7-277-ihRNA2 as AR knock-down group. Mammospheres formation results showed that 3.73% ± 0.32 of tumor cells formed mammospheres in MCF7-277-ihRNA2 group, compared with 2.03% ± 0.31 of those in MCF7-277-iluc group (*P* = 0.003, Figure 3C). CD44 expression was only detected in MCF7-277-ihRNA2 cells (Figure 3D), suggesting that these cells had the properties of tumor-initiating. Cells gathered from mammospheres were analyzed by flow cytometry assay, and results showed that tumor cells with CD44^+^/CD24^-/low^ phenotype increased significantly from mammospheres than those in control groups (Figure 3E), suggesting that breast tumor-initiating cells could be enriched by mammospheres cultivation .

### AR knocking down promoted tumorigenesis and metastasis capacity

**Figure 4:**
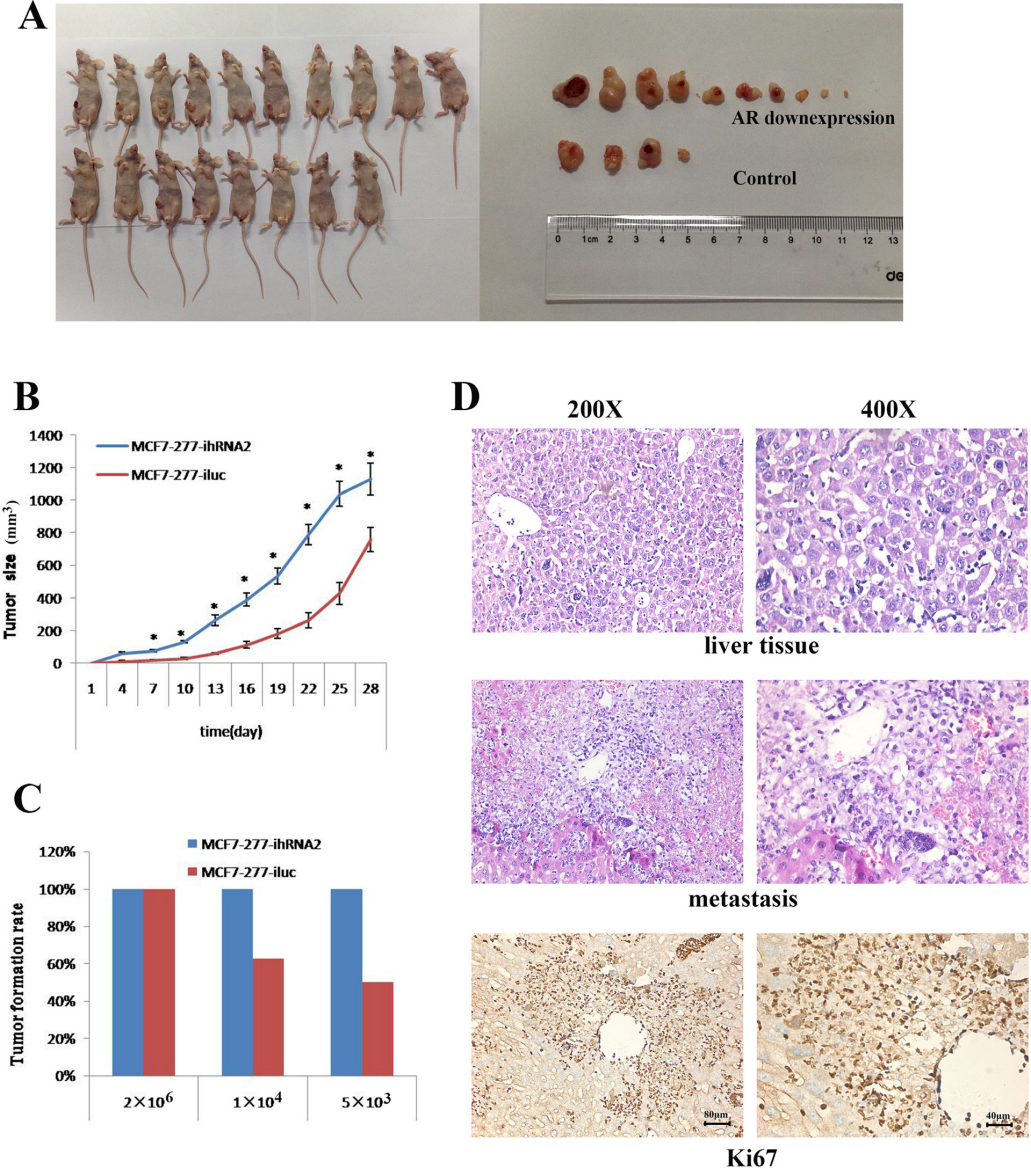
AR knockdown promoted tumorigenic capacity and metastasis in mice Tumors formation and tumors size was shown in AR knockdown and control groups (**A**). Tumor growth curves in MCF7-277-ihRNA2 and MCF7-277-iluc groups after tumor xenotransplantation (**B**). Tumor formation rates in MCF7-277-ihRNA2 and MCF7-277-iluc groups (**C**). HE staining of livers without or with metastasis. Immunohistochemical staining of Ki67 in metastatic liver tissue (**D**).

In order to analyze the effect of AR on BT-IC *in vivo*, we established a subcutaneous xenotransplanted tumor model in NOD/SCID mice with MCF7 cells in a gradually decreased cell density. There were significant differences of tumor volume between MCF7 expressing control and shRNA against AR groups. And tumors grew faster in MCF7-277-ihRNA2 group than in the control group (Figure 4A–4B). When we decreased the injected cell density, all mice receiving MCF7 cells expressing shRNA against AR still had tumorigenic growth while the tumor formation rate decreased significantly in control group (Figure 4C). In addition, liver metastasis was found in two of ten mice receiving MCF7-277-ihRNA2 cells with a cell density of 2 × 10^6^, but no metastasis was found in MCF7-277-iluc group. And metastatic cancer cells had high proliferation ability indicated by Ki67 staining (Figure 4D).

### AR transcriptionally regulated let-7a expression by activating let-7a promoter activity

**Figure 5:**
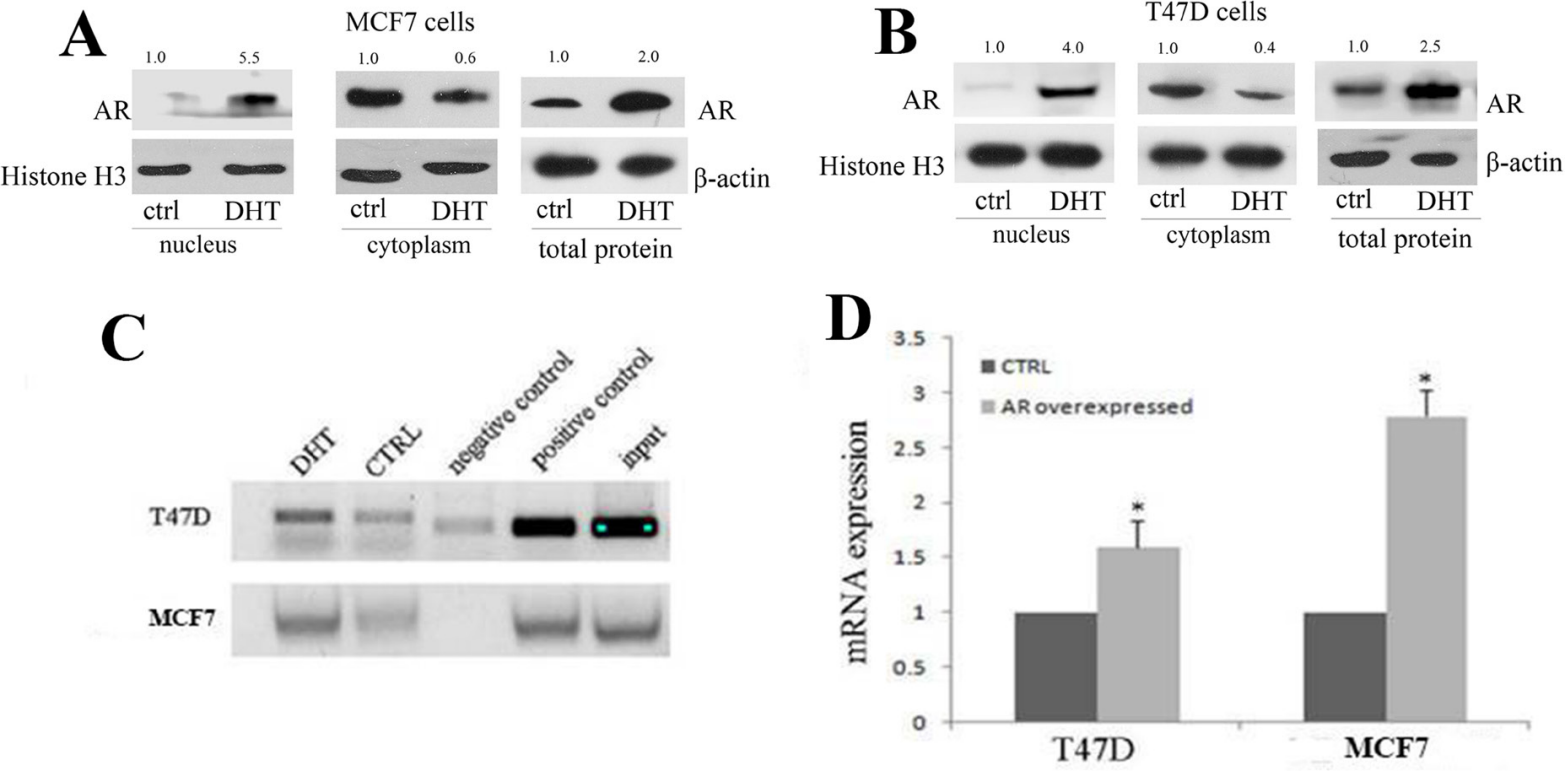
AR signaling transcriptionally mediated the expression of let-7a by activating let-7a promoter activity Western blot analysis showed AR expression of different location after DHT stimulation in MCF7 (**A**) and T47D (**B**) cells. Chromatin immunoprecipitation analysis in T47D and MCF7 cells (**C**). Luciferase analysis inT47D and MCF7 cells (**D**).

We then analyzed the mechanism of let-7a up-regulation by AR activation. DHT treatment induced AR imported into nucleus, indicated by western blot results in both MCF7 and T47D cell lines (Figure 5A and 5B). We inferred that androgen/AR signaling might transcriptionally mediate the regulation of let-7a in ER+AR+ breast cancer cells, just like the mechanism in ER-AR+ breast cancer cells [11]. As CHIP results shown (Figure 5C), cells treated with DHT induced an AR enriching on let-7a promoter region than those without DHT stimulation, which was confirmed by the following luciferase analysis. We constructed luciferase expression vectors of let-7a promoter based on androgen response element. The luciferase activity was higher in both AR over-expression MCF7 and T47D cells (Figure 5D), suggesting that AR could directly activate the let-7a promoter activity. These results suggested that the mechanism of androgen-AR signaling regulating let-7a was also reasonable in ER+/AR+ breast cancer cells.

## DISCUSSION

AR is a key transcription factor and mainly functions in nucleus, when being activated by androgens. Studies suggest AR play an important role in breast cancer development [18], in part via the role of let-7 up-regulation induced by AR activation [11]. Cancers may arise from rare self-renewing tumor-initiating cells (T-IC). In prostate cancer, AR expression is previously described to be associated with T-IC, and AR signal can influence T-IC [4]. Let-7 is the earliest discovered miRNAs [13], which may inhibit multiple BT-IC stem cell-like properties by silencing more than one target [16]. Let-7a is one of the members of let-7 family and usually selected as the representative [14, 15]. At present, few are known on the relationship among AR, let-7a and the self-renewing BT-IC in breast cancer, and also their interactions.

In breast cancer tissue, we identified that AR expression was positively correlated with let-7a expression, but negatively with CD44^+^/CD24^-/low^ phenotype of cancer cells. We suppose that AR, let-7a and BT-IC may affect the progression of breast cancer together. AR expression in primary tumors is considered as a better prognostic indicator in a majority of previous studies [2, 3, 19]. T-ICs are characteristic by self-renewal, rapid proliferation, differentiated tumor cells and drug resistance, which may be responsible for the recurrence and metastasis of breast cancer [7, 10, 20]. The present study also indicated that patients with CD44^+^/CD24^-/low^ phenotype tumors had worse outcome. Let-7a expression is decreased in breast cancer, and the loss of let-7 expression is correlated with the development of poorly differentiated and aggressive cancers, which is considered to function as tumor suppressors [16, 21–23]. Overexpression of let-7a inhibits the ability of BT-ICs and reduces cells proliferation [24]. However, one report states the oncogenic activity of let-7a [25]. Therefore, more studies should be carried out to analyze the role of let-7a in breast cancer.

AR activation can inhibit estrogen-stimulated proliferation [26], and AR-induced cell apoptosis is also detected in ER+ breast cancer cells [27]. We found that androgen-induced AR activating signal affects cells behavior both in ER+AR+ breast cancer cells, and also in ER-AR+ breast cancer cells [11]. It is reported that AR/ER ratio can influence cells response to androgen, and increased ratio of AR/ER inhibits E2-induced proliferation [28], which further illustrate that AR may function in breast cancer dependent on ER expression. And some co-activators have an important role in the phenotype of cells with different AR/ER ratios. From the present and our previous study, we concluded that the inhibition of some biological behavior of breast cancer cells induced by AR signaling might be via let-7a up-regulation in part [11]. However, in prostate cancer, let-7 suppresses AR expression and activity, which in turn leads to decreased cell proliferation and tumor growth [29, 30]. Therefore, we suspects that AR and let-7a may have complicated associations depending on the different context. And more studies should be carried out to analyze whether let-7a will down-regulate AR expression and affect androgen-AR signaling in breast cancer cells.

The positive association between the CD44^+^/CD24^-/low^ presence and AR expression was more obvious in ER+ breast cancers, which was consistent with our *in vitro* results. AR activation could decrease the mammospheres formation, while AR knockdown increase in ER+AR+ breast cancer cells when they were cultured in suspension. The flow cytometry assay results confirmed that decreased AR expression significantly increased the percentage of MCF7 cells with CD44^+^/CD24^-/low^ phenotype whether in attachment or in suspension (Figure 5). And CD44 expression could be detected in AR knockdown cancer cells, but not in control group, indicated by western blot results. There are several publications that suggest AR expression correlates significantly with cancer stem cells mainly in prostate cancers [31, 32], but few is known about their correlations in breast cancers.. The important T-IC features are efficient xenograft formation and metastases initiation. In Al-Hajj’s study [7], as few as 1000 cells with CD44^+^/CD24^-/low^ phenotype can give rise to tumors in all cases, while up to 2 × 10^4^ CD44^+^/24^+^ cells fail to form tumors, which suggests that cancer cells with CD44^+^/CD24^-/low^ phenotype have the tumorigenic capability upon serial xenotransplantation *in vivo*. We subcutaneously injected MCF7 cells of different AR expression into NOD/SCID mice with a gradually decreased cell density. AR knockdown could promote tumor formation and growth in the mice receiving cancer cells even with a decreased cell number, suggesting an enhanced tumorigenic capability. In addition, two mice developed into liver metastasis in AR knockdown group, but no in controls. No lung metastasis was observed in all mice. It is suspected that the heterogeneity of breast cancer cells contributes to the difference, and more studies should be performed to analyze this phenomenon.

AR is a key transcription factor and androgens induce AR translocate into the nucleus, where mainly function. Several recent studies state that AR can directly regulate the transcription of miRNAs [33, 34] , which is consistent to our study. Our group indicated that AR imported into the nucleus and up-regulated let-7a expression by binding to the androgen response elements of let-7a promoter region in breast cancer cells, upon androgen activation [11, 35]. We speculated that the mechanism of AR affecting breast T-IC might be partially via transcriptional regulation of let-7a, since let-7a overexpression inhibits T-IC capabilities in ER+ breast cancers [24]. However, some additional transcription factors or co-regulating proteins may affect breast T-IC in ER- breast cancers.

In conclusion, our findings indicated that DHT-induced AR activation played a critical role in breast cancer, which was not only correlated with let-7a expression, but also related to BT-IC. DHT treatment induced AR translocated into the nucleus and transcriptionally up-regulated let-7a expression directly, and then decreased cells proliferation, migration, invasion, and self-renewal capacities. In addition, AR knockdown promoted tumor-formation and metastasis capacity, even when cancer cells were xenotransplanted into the mice with a decreased cell density. Patients with enhanced AR or let-7a expression predicted a better prognosis, while the tumor cells with CD44^+^/CD24^-/low^ phenotype predicted a worse prognosis index. A better understanding of the effect and mechanism of AR-let-7a signalling and breast T-IC will lead to new therapeutic strategies.

## MATERIALS AND METHODS

### Clinical specimens

One hundred and sixty-five invasive breast cancer tissues were selected, which were diagnosed between January 2005 and May 2005. These patients were registered in the Department of Breast Cancer Pathology and Research Laboratory, Tianjin Medical University Cancer Instituted and Hospital, Tianjin, China. All of these selected patients signed informed consent before the examination of the specimens, and the research was approved by the Institutional Ethic Committee of Tianjin Medical University Cancer Instituted and Hospital. Patients with bilateral primary breast cancers and those who received preoperative treatment were excluded, and their clinicopathological data were available. Participants in the cohort were women ranging in age from 32 to 78 years (median 51 years).

### Immunohistochemical assay

Immunohistochemistry was performed using the Double-Staining Methods based on Streptavidin Alkaline Phosphatase and Streptavidin Peroxidase labeling techniques (KIT-9999, MXB-China). All of the procedures were carried out according to the manufacturer's instructions. Sections were stained with anti-rabbit CD44 antibody (1:100, ZA-0537, ZSGB, China) and anti-mouse CD24 antibody (1:100, ab31622, Abcam, USA). To control the reliability of the CD44 and CD24 double-staining, single staining with CD44 and CD24 was done. Immunohistochemistry for AR (1:200, ZA-0554, ZSGB, China) and Ki67 (working solution, ZA-0502, ZSGB, China) were carried out as previously reported [36]. Sections were incubated with goat serum for negative controls of immunoreactions.

### In situ hybridization(ISH) and evaluation of the staining

The MicroRNA ISH Detection Kit III (MK1031, BOSTER, China) and miRNAlet7a probes (38468-05, Exiqon, Denmark) were used for ISH in the present study. All procedures were carried out according to the manufacturer’s instructions. Briefly, formalin-fixed paraffin-embedded breast cancer tissue were cut into 4-µm sections and deparaffinized. Tissue permeabilization was used by proteinase. After pre-hybridization, sections were incubated with the hybridization mix and hybridized for overnight at 45 °C. For signal detection, anti-digoxigenin-alkaline phosphatase were used as the primary antibody, and the slides were then subsequently incubated with 5-bromo-4-chloroindo-3-yl-phosphate/nitro blue tetrazolium (BCIP/NBT) solution Counterstaining was performed by Nuclear Fast Red. The stained sections were then scored for expression of let7a miRNA under the microscopy. For the negative control, the hybridization solution was replaced with the pre-hybridization solution.

### Cells culture

MCF-7 and T47D human breast cancer cell lines were obtained from American Type Culture Collection (ATCC, USA), and cultured in DMEM medium (Gibco, USA), which contained 10% fetal bovine serum (FBS, Gibco, USA) and 1% penicillin/streptomycin (Life Technologies, USA), and cultured in a incubator with 5% CO_2_ at 37°C.

### Lentiviral vector production

Oligos encoding shRNA specific for AR were ligated into pSUPER.reto.puro, and the fragment containing the H1 promoter and hairpin sequences was subcloned into the lentiviral 277 vectors. The shRNA target sequences were as follows: AR-ihRNA1: CGACTACTACAACTTTCCA; AR-ihRNA2: AATGTTATGAAGCAGGGAT. The plasmids were used to produce lentivirus in 293T competent cells with the packaging plasmids including pMD2.BSBG, pMDLg/pRRE and pRSV-REV.

### Transfection

MCF-7 and T47D cancer cells were transfected with let-7a antisense oligonucleotide (ASO) and the control ASO (Coralville, IA, USA) using Lipofectamine 2000 (Invitrogen, USA). According to the previously reported protocol [11], the oligonucleotides complementary to let-7a and control ASO sequences were as follows: let-7a ASO, 5’-AACTATACAACCTACTACCTCA-3’, and control ASO, 5’-GTGGATATTGTTGCCATCA-3’. Cells were treated with dihydrotestosterone (DHT) for additional 48h after being cultured in DMEM containing 10% charcoal stripped serum for 24 h. MCF-7 cells were infected with lentivirus of AR-ihRNA1 and AR-ihRNA2 constructed by 277 vectors, and selected with G418 (300µg/ml) to generate a stable AR knockdown cell line.

### Real-time reverse transcription PCR (real-time RT-PCR)

Total RNA was extracted from cells with TRIzol (Invitrogen, USA). RT-PCR system (TaKaRa, Japan) was used to reverse RNA, and 1 ul cDNA sample was quantified using primers with SYBR Green PCR Master mix (TaKaRa, Japan) by real-time PCR. Let-7a specific primers were used as previously reported [11]. All analyses were performed in triplicate.

### Western blot assay

Cells were lysed in RIPA buffer containing phosphatase inhibitor after washed by ice-cold PBS. Western blotting was performed as previously described [11]. Antibodies used in the present study were as follows: anti-AR (1:1000, ab212498, Abcam, USA), anti- CD44 (1:1000, ab51037, Abcam, USA), anti p-STAT3 (1:1000, ab32143, Abcam, USA), anti-STAT3 (1:5000, ab119352, Abcam, USA), anti-β-actin (1:1000, 3700, Cell Signaling, USA), and anti-Histone H3 (1:1000, 9728, Cell Signaling, USA) antibodies.

### IL-6 enzyme-linked immunosorbent assay (ELISA)

According to the manufacturer’s protocol, culture supernatants of cells were harvested and concentrations of IL-6 proteins were evaluated by ELISA kits (R&D Systems, China). Briefly, protein was pipetted into a microplate containing IL-6 antibody and incubated for 2 h; IL-6 antibody with enzyme-linked was added and incubated for 1 h; added a substrate solution and incubated for 15 min. When positive control wells emerged blue color, stopping reagent was added to stop the reaction. Then samples were detected at 450 nm absorbance.

### Flow cytometry analysis

5 × 10^5^ cells were collected and fixed with methanol. Then cells were stained by propidium iodide (Sigma-Aldrich); finally all samples were analyzed by flow cytometer. Proliferation index (PI) was calculated as follows: PI = (S+G2/M)/G1. Cells were incubated with PE-labeled anti-CD24 and FITC-labeled anti-CD44 (Miltenyi Biotec, Germany) and then analyzed by flow cytometer for cell surface staining.

### MTT, transwell and scratch assay

Cell proliferation and viability were measured by MTT assay. The procedure was carried out as previously reported [11]. For transwell assay, 24-well transwell chamer plates were used. 1 × 10^5^ cells suspended with serum-free medium were seeded into the upper chamber (8 μm pore size, Corning, USA), which was coated with Matrigel (BD, USA) diluted by serum-free medium. Complete medium was added into the bottom chamber. The invaded cells were stained with Gimesa staining solution after 24 h and counted. For scratch assays, cells (5 × 10^5^) were plated into cell culture dishes and cultured in serum free DMEM. The monolayer was wounded by a yellow pipette tip, and then removed the cellular debris with PBS. The exact initial wound areas were taken photographs every 2 days.

### Mammosphere Culture

1000 cells per well were cultured in suspension to generate mammospheres in specific 6-well Ultra-Low attachment plates (Corning, USA), and cultured with DMEM/F12 medium (Gibco, USA) without serum, supplemented with 20 ng/mL EGF (BD, USA), B27 (1:250, Invitrogen, USA), 10 ng/mL bFGF (BD, USA), as well as 0.4 ug/mL insulin (Sigma, USA). Medium was exchanged every 3 days, and the mammospheres were observed every 3 days.

### Tumor implantation

Indicated numbers of MCF7 cells were injected into the mammary fat pads of NOD/SCID mice (6–8 weeks, female). Before injection, all mice were treated with oral estrogen for two weeks. Within the following 30 days, all mice were examined for tumor formation by palpation. The volume calculated as follows: Volume (mm^3^) = L.W^2^/2, and tumor size was measured every 3 days.

### Chromatin immunoprecipitation

Cells were fixed using 1% formaldehyde for cross-linking the DNA and protein. ChIP was performed according to the ChIP Assay Kit’s protocols (17-371, Upstate, USA). Briefly, sonicated nucleoprotein was incubated with 10µg of AR antibody (ab74272, Abcam, USA). 1µg polymerase antibody was replaced as positive control and 1µg normal mouse IgG as negative control. Sixty microliters of protein G-Sepharose beads was added for every reaction. The Sepharose immunocomplex slurries were incubated at 65°C overnight to reverse cross-links and were then RNase treated, proteinase K treated, deproteinized, and precipitated as described previously [37]. Immunoprecipitated and input DNA fractions were analyzed by PCR. In order to investigate the binding of AR and androgen response element (ARE) regions of the let-7a promoter, we first analyzed the potential ARE in Let-7a promoter regions by PROMO soft, and then designed the specific primers for the 251bp DNA ARE fragment in let-7a promoter region. They were as follows: let-7a-ARE-F: TTTTACATTGGGCATAGCCG; let-7a-ARE-R: TAGGCATTTGGAAGTTGGAC. The PCR products were detected by 1% agarose gel electrophoresis and analyzed with PCR Gel Image Analysis System.

### Luciferase assay

Androgen response element of the 3200bp sequences upstream let-7a 5’ end region was cloned into the luciferase reporter expression vector (pEZX-PG04, Genecopoeia, USA) AR transcript 1A CDS region was cloned into the pReceiver-M12 vectors (EX-M12, GeneCopoeia, USA). MCF7 and T47D cells were seeded into the 6-well plates at a density of (2-4) × 10^5^cells/well. Luciferase let-7a promoter expression and AR over-expressing plasmids were transiently co-transfected into the cells using Lipofectamine 2000 (Invitrogen). Then the luciferase activities were evaluated by a Secrete-Pair Dual Luminescence Assay Kit (Genecopoeia), following the manufactures’ instructions at the time of 24-48 hours after transfection. Luciferase activities were detected with a luminometer, and then normalized by luciferase activity in luciferase reporter.

### Statistical analysis

Statistical analysis was performed by SPSS 19.0 statistical software. The data were reported as the Means ± SD. Statistical analysis was carried out using one-way ANOVA in comparison with he corresponding controls. Differences between AR, let-7a, CD44^+^/CD24^-/low^ and clinicopathological characteristics were analyzed via the Chi-square test. The association among AR, let-7a and CD44^+^/CD24^-/low^ was tested using Spearman rank correlation with Pearson’s test. Based on the Kaplan-Meier method, disease-free survival (DFS) and overall survival (OS) curves were generated. Differences between the curves were evaluated by the log-rank test. Probability values of < 0.05 were regarded as statistically significant.

## References

[1] Gonzalez LO, Corte MD, Vazquez J, Junquera S, Sanchez R, Alvarez AC, Rodriguez JC, Lamelas ML, Vizoso FJ (2008). Androgen receptor expresion in breast cancer: relationship with clinicopathological characteristics of the tumors, prognosis, and expression of metalloproteases and their inhibitors. BMC Cancer.

[2] Hickey TE, Robinson JL, Carroll JS, Tilley WD (2012). Minireview: The androgen receptor in breast tissues: growth inhibitor, tumor suppressor, oncogene?. Mol Endocrinol.

[3] Yu Q, Niu Y, Liu N, Zhang JZ, Liu TJ, Zhang RJ, Wang SL, Ding XM, Xiao XQ (2011). Expression of androgen receptor in breast cancer and its significance as a prognostic factor. Ann Oncol.

[4] Qin J, Liu X, Laffin B, Chen X, Choy G, Jeter CR, Calhoun-Davis T, Li H, Palapattu GS, Pang S, Lin K, Huang J, Ivanov I (2012). The PSA(-/lo) prostate cancer cell population harbors self-renewing long-term tumor-propagating cells that resist castration. Cell Stem Cell.

[5] Lee SO, Ma Z, Yeh CR, Luo J, Lin TH, Lai KP, Yamashita S, Liang L, Tian J, Li L, Jiang Q, Huang CK, Niu Y (2013). New therapy targeting differential androgen receptor signaling in prostate cancer stem/progenitor vs. non-stem/progenitor cells. J Mol Cell Biol.

[6] O’Brien CA, Pollett A, Gallinger S, Dick JE (2007). A human colon cancer cell capable of initiating tumour growth in immunodeficient mice. Nature.

[7] Al-Hajj M, Wicha MS, Benito-Hernandez A, Morrison SJ, Clarke MF (2003). Prospective identification of tumorigenic breast cancer cells. Proc Natl Acad Sci U S A.

[8] Thike AA, Yong-Zheng Chong L, Cheok PY, Li HH, Wai-Cheong Yip G, Huat Bay B, Tse GM, Iqbal J, Tan PH (2014). Loss of androgen receptor expression predicts early recurrence in triple-negative and basal-like breast cancer. Mod Pathol.

[9] Patrawala L, Calhoun T, Schneider-Broussard R, Zhou J, Claypool K, Tang DG (2005). Side population is enriched in tumorigenic, stem-like cancer cells, whereas ABCG2+ and ABCG2- cancer cells are similarly tumorigenic. Cancer Res.

[10] Ponti D, Costa A, Zaffaroni N, Pratesi G, Petrangolini G, Coradini D, Pilotti S, Pierotti MA, Daidone MG (2005). Isolation and *in vitro* propagation of tumorigenic breast cancer cells with stem/progenitor cell properties. Cancer Res.

[11] Lyu S, Yu Q, Ying G, Wang S, Wang Y, Zhang J, Niu Y (2014). Androgen receptor decreases CMYC, KRAS expression by upregulating let-7a expression in ER-, PR-, AR+ breast cancer. Int J Oncol.

[12] Lai EC (2002). Micro RNAs are complementary to 3’ UTR sequence motifs that mediate negative post-transcriptional regulation. Nat Genet.

[13] Medina PP, Slack FJ (2008). microRNAs and cancer: an overview. Cell Cycle.

[14] Muller DW, Bosserhoff AK (2008). Integrin beta 3 expression is regulated by let-7a miRNA in malignant melanoma. Oncogene.

[15] Sureban SM, May R, Ramalingam S, Subramaniam D, Natarajan G, Anant S, Houchen CW (2009). Selective blockade of DCAMKL-1 results in tumor growth arrest by a Let-7a MicroRNA-dependent mechanism. Gastroenterology.

[16] Yu F, Yao H, Zhu P, Zhang X, Pan Q, Gong C, Huang Y, Hu X, Su F, Lieberman J, Song E (2007). let-7 regulates self renewal and tumorigenicity of breast cancer cells. Cell.

[17] Honeth G, Bendahl PO, Ringner M, Saal LH, Gruvberger-Saal SK, Lovgren K, Grabau D, Ferno M, Borg A, Hegardt C (2008). The CD44+/CD24- phenotype is enriched in basal-like breast tumors. Breast Cancer Res.

[18] Agrawal AK, Jelen M, Grzebieniak Z, Zukrowski P, Rudnicki J, Nienartowicz E (2008). Androgen receptors as a prognostic and predictive factor in breast cancer. Folia Histochem Cytobiol.

[19] Peters KM, Edwards SL, Nair SS, French JD, Bailey PJ, Salkield K, Stein S, Wagner S, Francis GD, Clark SJ, Brown MA (2012). Androgen receptor expression predicts breast cancer survival: the role of genetic and epigenetic events. BMC Cancer.

[20] Abraham BK, Fritz P, McClellan M, Hauptvogel P, Athelogou M, Brauch H (2005). Prevalence of CD44+/CD24-/low cells in breast cancer may not be associated with clinical outcome but may favor distant metastasis. Clin Cancer Res.

[21] Johnson SM, Grosshans H, Shingara J, Byrom M, Jarvis R, Cheng A, Labourier E, Reinert KL, Brown D, Slack FJ (2005). RAS is regulated by the let-7 microRNA family. Cell.

[22] Mayr C, Hemann MT, Bartel DP (2007). Disrupting the pairing between let-7 and Hmga2 enhances oncogenic transformation. Science.

[23] Liu K, Zhang C, Li T, Ding Y, Tu T, Zhou F, Qi W, Chen H, Sun X (2015). Let-7a inhibits growth and migration of breast cancer cells by targeting HMGA1. Int J Oncol.

[24] Xu C, Sun X, Qin S, Wang H, Zheng Z, Xu S, Luo G, Liu P, Liu J, Du N, Zhang Y, Liu D, Ren H (2015). Let-7a regulates mammosphere formation capacity through Ras/NF-kappaB and Ras/MAPK/ERK pathway in breast cancer stem cells. Cell Cycle.

[25] Brueckner B, Stresemann C, Kuner R, Mund C, Musch T, Meister M, Sultmann H, Lyko F (2007). The human let-7a-3 locus contains an epigenetically regulated microRNA gene with oncogenic function. Cancer Res.

[26] Ortmann J, Prifti S, Bohlmann MK, Rehberger-Schneider S, Strowitzki T, Rabe T (2002). Testosterone and 5 alpha-dihydrotestosterone inhibit *in vitro* growth of human breast cancer cell lines. Gynecol Endocrinol.

[27] Kandouz M, Lombet A, Perrot JY, Jacob D, Carvajal S, Kazem A, Rostene W, Therwath A, Gompel A (1999). Proapoptotic effects of antiestrogens, progestins and androgen in breast cancer cells. J Steroid Biochem Mol Biol.

[28] Szelei J, Jimenez J, Soto AM, Luizzi MF, Sonnenschein C (1997). Androgen-induced inhibition of proliferation in human breast cancer MCF7 cells transfected with androgen receptor. Endocrinology.

[29] Nadiminty N, Tummala R, Lou W, Zhu Y, Shi XB, Zou JX, Chen H, Zhang J, Chen X, Luo J, deVere White RW, Kung HJ, Evans CP (2012). MicroRNA let-7c is downregulated in prostate cancer and suppresses prostate cancer growth. PLoS One.

[30] Nadiminty N, Tummala R, Lou W, Zhu Y, Zhang J, Chen X, deVere White RW, Kung HJ, Evans CP, Gao AC (2012). MicroRNA let-7c suppresses androgen receptor expression and activity via regulation of Myc expression in prostate cancer cells. J Biol Chem.

[31] Collins AT, Berry PA, Hyde C, Stower MJ, Maitland NJ (2005). Prospective identification of tumorigenic prostate cancer stem cells. Cancer Res.

[32] Gu G, Yuan J, Wills M, Kasper S (2007). Prostate cancer cells with stem cell characteristics reconstitute the original human tumor *in vivo*. Cancer Res.

[33] Shi XB, Xue L, Yang J, Ma AH, Zhao J, Xu M, Tepper CG, Evans CP, Kung HJ, deVere White RW (2007). An androgen-regulated miRNA suppresses Bak1 expression and induces androgen-independent growth of prostate cancer cells. Proc Natl Acad Sci U S A.

[34] Takayama K, Tsutsumi S, Katayama S, Okayama T, Horie-Inoue K, Ikeda K, Urano T, Kawazu C, Hasegawa A, Ikeo K, Gojyobori T, Ouchi Y, Hayashizaki Y (2011). Integration of cap analysis of gene expression and chromatin immunoprecipitation analysis on array reveals genome-wide androgen receptor signaling in prostate cancer cells. Oncogene.

[35] Claessens F, Verrijdt G, Schoenmakers E, Haelens A, Peeters B, Verhoeven G, Rombauts W (2001). Selective DNA binding by the androgen receptor as a mechanism for hormone-specific gene regulation. J Steroid Biochem Mol Biol.

[36] Liu J, Liu X, Feng X, Liu J, Lv S, Zhang W, Niu Y (2015). C-kit overexpression correlates with KIT gene copy numbers increases in phyllodes tumors of the breast. Breast Cancer Res Treat.

[37] Liu Z, Garrard WT (2005). Long-range interactions between three transcriptional enhancers, active Vkappa gene promoters, and a 3’ boundary sequence spanning 46 kilobases. Mol Cell Biol.

